# Comparative Genomic Analysis of *Lactiplantibacillus plantarum*: Insights into Its Genetic Diversity, Metabolic Function, and Antibiotic Resistance

**DOI:** 10.3390/genes16080869

**Published:** 2025-07-24

**Authors:** Ruiqi Li, Chongpeng Bi

**Affiliations:** 1 College of Veterinary Medicine, Northeast Agricultural University, Harbin 150030, China; 13034591915@163.com; 2 College of Animal Science and Technology, Northeast Agricultural University, Harbin 150030, China

**Keywords:** *Lactiplantibacillus plantarum*, genomic analysis, metabolic function, probiotic mechanism, biosafety

## Abstract

**Background/Objectives**: *Lactiplantibacillus plantarum* is widely utilized in the fermentation industry and offers potential health benefits. However, large-scale comparative genomic analyses aimed at exploring its metabolic functions and conducting safety assessments are still lacking. **Methods**: In this study, we performed a comparative genomic analysis of 324 *L. plantarum* strains sourced from various origins and geographical locations. **Results**: The results revealed that *L. plantarum* possesses a total of 2403 core genes, of which 12.3% have an unknown function. The phylogenetic analysis revealed a mixed distribution from various origins, suggesting complex transmission pathways. The metabolic analysis demonstrated that *L. plantarum* strains can produce several beneficial metabolites, including lysine, acetate, and riboflavin. Furthermore, *L. plantarum* is highly capable of degrading various carbohydrates and proteins, increasing its adaptability. Further, we profiled the antimicrobial peptides (AMPs) in the genomes of *L. plantarum*. We identified a widely distributed AMP and its variants, presenting in a total of 280 genomes. In our biosafety assessment of *L. plantarum*, we identified several antibiotic resistance genes, such as *Tet(M)*, *ANT(6)-Ia*, and *mdeA*, which may have potential for horizontal gene transfer within the *Lactobacillaceae* family. **Conclusions**: This study provides genomic insights into the genetic diversity, metabolic functions, antimicrobial properties, and biosafety of *L. plantarum*, underscoring its potential applications in biotechnology and environmental adaptation.

## 1. Introduction

*Lactiplantibacillus plantarum*, formerly known as *Lactobacillus plantarum*, is a species of lactic acid bacteria that can be isolated from a variety of sources, including humans, animals, grass silage, kimchi, pickled cabbage, cheese, etc. [1]. This bacterium can metabolize pentose and hexose sugars to produce lactic acid, carbon dioxide, and either acetate or ethanol [2]. Thus, it plays a crucial role in the fermentation of foods such as sauerkraut, kimchi, pickles, and dairy products [3]. In addition to being an important component in food fermentation, *L. plantarum* also functions as a probiotic, enhancing human health. For example, *L. plantarum* L168 has been shown to have several beneficial properties, including restoring the structure of the gut microbiota, modulating the concentration of the neurotransmitter serotonin, and improving impaired behavior [4].

The widespread application of *L. plantarum* in the food industry and its potential as a probiotic necessitate a comprehensive understanding of its genetic diversity, phylogenetic relationships, and functional characteristics. In recent years, advancements in next-generation sequencing technologies have facilitated numerous genomic studies of *L. plantarum* strains, providing deep insights. In the genome of *L. plantarum* LP-F1, a total of 130 enzymes related to carbohydrate metabolism were identified, highlighting its significant capacity for carbohydrate utilization [5]. A genomic analysis of *L. plantarum* L125 revealed the presence of an incomplete plataricin gene cluster, with no bacteriocin-like activity detected [6]. Additionally, a genomic analysis of *L. plantarum* BRD3A suggested its potential to produce bacteriocins such as plantaricin E, lantaricin F, and enterocin X [7]. Furthermore, the genomic analyses also indicated a higher prevalence of antimicrobial resistance genes in 212 *L. plantarum* genomes, particularly those associated with tetracycline-resistance [8].

With the accumulation of *L. plantarum* genomes, a comparative genomic analysis would be helpful in providing a comprehensive overview of their characteristics. Garcia-Gonzalez and colleagues conducted a comparative genomic analysis of 42 *L. plantarum* strains to gain insights into their probiotic properties [9]. Similarly, Evanovich and colleagues also performed a study that did not identify any antibiotic resistance genes (ARGs) in the *L. plantarum* genome [10]. A recent investigation evaluated the antibiotic resistance patterns of *L. plantarum* strains, revealing the presence of resistance to ampicillin [11]. Martino and colleagues noted a loose connection between the distribution and source of *L. plantarum* [12], which may be attributed to the insufficient number of genomes available. With the increasing number of genomes, there is an opportunity for large-scale comparative genomic analyses to reevaluate these relationships.

In this study, we collected a total of 324 complete genomes of *L. plantarum* strains from NCBI, which were isolated from various sources across six continents. A comprehensive genomic analysis was conducted to explore the genomic characteristics, phylogenetic relationships, metabolic functions, and biosafety of these strains. The findings of this study provide valuable insights into the probiotic properties and biosafety of *L. plantarum*, offering a scientific basis for its application in the food industry and probiotic development.

## 2. Materials and Methods

### 2.1. Collection of Microbial Genomes

To better understand the genomic characteristics of *L. plantarum*, a total of 324 complete genomes (as of 29 March 2025) were deposited in the National Center for Biotechnology Information (NCBI, Supplementary Table S1). To further confirm the quality of these genomes, we utilized GTDB-Tk v2.4.0 [13] and checkM2 v1.0.1 [14] to verify the taxonomic classification and genome quality, respectively. The phylogenetic relationships among different strains and plasmids were calculated using average nucleotide identity (ANI) with pyani v0.2.3 [15]. These genomes were subsequently annotated with Prokka v1.14.6 [16], which identified open reading frames for downstream analyses.

### 2.2. Construction of the Phylogenetic Tree

A pan-genome analysis of the 324 genomes was conducted using Panaroo v1.14.6 [17] to explore the genetic diversity and identify core and accessory genes within the *L. plantarum* strains. The functional annotations of the core genes were performed using eggNOG v2.1.12 [18].

We then constructed the phylogenetic tree using IQ-TREE2 v2.1.4-beta [19], which is based on the core genome alignment with the parameters of “-m TEST -B 1000 -bnni”. The phylogenetic tree was further clustered using fastBAPS [20].

### 2.3. Prediction of Metabolic Profiles

Functional annotations of the *L. plantarum* genomes were conducted using the metabolic-G.pl program in METABOLIC v4.0 [21], which integrates the Kyoto Encyclopedia of Genes and Genomes (KEGG), TIGRfam, Pfam, dbCAN2, and MEROPS databases. This process includes a protein motif validation step to evaluate the presence of metabolic pathways on the basis of KEGG modules. The annotations were subsequently manually reviewed for the presence of KEGG modules, carbohydrate-active enzymes (CAZymes), peptidases, and their inhibitors.

### 2.4. In Silico Analysis of Virulence Genes and Antimicrobial Resistance and Their Associated Mobile Genetic Elements (MGEs)

Virulence genes were annotated using ABRicate (https://github.com/tseemann/abricate (accessed on 6 March 2025)) against the Virulence Factor Database using the default parameters [22]. ARGs were annotated using the Comprehensive Antibiotic Resistance Database (CARD, v6.0.2) [23]. To reduce the number of potential false positives, only perfect and strict hits were retained. To establish links between these genes and MGEs, a detailed analysis of the *L. plantarum*-associated prophage fragments and plasmids was conducted. First, we used geNomad v1.5.2 [24] and VITAP v1.7.1 [25] to identify the prophage fragments and perform the taxonomic classification. We then typed these plasmids using the function “mob_typer” embedded in the software MOB-suite v3.1.0 [26].

### 2.5. In Silico Analysis of Antimicrobial Peptides (AMPs)

Macrel v1.3.0 [27] was run directly on the genomes to identify the AMPs. To assess the novelty of the predicted AMPs, we manually constructed an AMP database comprising 44,406 AMPs sourced from five existing AMP databases [28,29,30,31,32]. Subsequently, the identified AMPs were queried against the curated AMP database with blastp v2.10.0+ [33]. Nonredundant AMP sequences were then clustered using CD-HIT v4.8.1 [34], with an identity of over 70%. The amino acid sequences of AMPs, which belong to the same cluster, were aligned using MUSCLE v3.8.31 with the default parameters [35]. The alignments were then used to construct phylogenetic trees using IQ-TREE2. To better understand the potential effects of the changes to the AMP sequences, APEX [36] was used to predict the species-specific antimicrobial activities of the AMPs, which were measured by the minimum inhibitory concentration (MIC) against 34 type strains.

### 2.6. Statistics and Visualization

The data in this study were expressed as the mean ± SD. The phylogenetic trees were visualized using iTOL v7 [37]. Circular genome maps of ARG-carrying plasmids were visualized using Proksee [38]. The other visualizations were constructed using R software v4.3.1, primarily with the pheatmap v1.0.12 [39] and ggplot2 v3.4.4 [40] packages. Pairwise comparisons between different groups were performed using the “pairwise.wilcoxon.test” function in R.

## 3. Results

### 3.1. Strain Information and Genome Characteristics

In this study, we collected a total of 324 complete *L. plantarum* genome sequences from NCBI (Supplementary Table S1). These strains were isolated from a variety of sources, including human-related samples (*n* = 62), fermented foods (*n* = 138), animal-related samples (*n* = 56), plants (*n* = 26), the environment (*n* = 7), and other sources (*n* = 35). The samples were distributed across six continents: Asia, Europe, North America, South America, Oceania, and Africa. These genome sizes ranged from 2,793,376 bp to 3,687,306 bp, with an average length of 3,305,355 bp. The genomes contained an average of 2.9 ± 2.8 plasmids, accounting for an average of 101,908 ± 98,232 bp.

The pan-genome analysis revealed that these genomes contained a total of 12,416 genes, of which 2403 were classified as core genes, including both core genes and soft-core genes (Table 1). The five most common COG categories of the core genes were classified as the following: [S] function unknown (12.3%); [K] transcription (6.0%); [E] amino acid transport and metabolism (5.5%); [J] translation, ribosomal structure, and biogenesis (4.6%); and [G] carbohydrate transport and metabolism (4.5%).

### 3.2. Phylogenetic Analysis Reveals Complex Transmission Pathways

**Figure 1:**
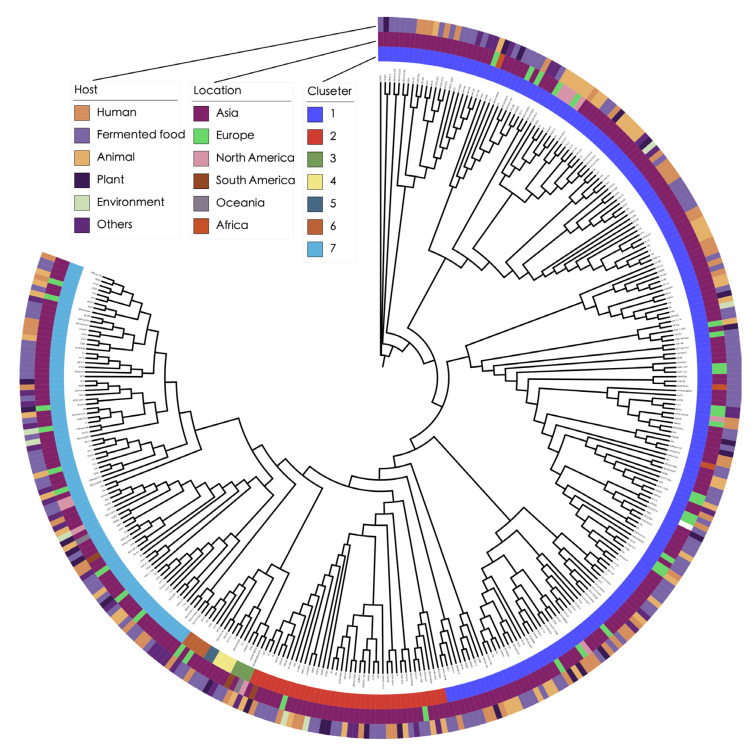
Phylogenetic tree constructed based on the core genomes of 324 *L. plantarum* strains. From the inner to outer, the three rings represent different clusters, the location, and the host of strains.

To investigate the phylogenetic relationships among the *L. plantarum* strains, a phylogenetic tree was constructed on the basis of the core genome, and the strains were grouped into seven different clusters (Figure 1). Three larger clusters were observed in the phylogenetic tree, consisting of 188 genomes in cluster 1, 82 genomes in cluster 7, and 39 genomes in cluster 2. From a host perspective, these three larger clusters included the strains from all five specific hosts included in this study, indicating no preference for any particular host (Supplementary Figure S1). From a geographical perspective, strains from other continents were distributed sporadically across the three larger clusters. This suggests a wider distribution of these genetically related strains. Additionally, there were no obvious associations between the host and the cluster, potentially because of the frequent transmission among various origins.

### 3.3. Key Metabolic Functions Revealed by the Presence of KEGG Modules in L. plantarum

Different strains of the same species may exhibit different metabolic functions and modulate host physiology by producing various metabolites. To better understand the potential production of metabolites, we further explored their metabolic functions by analyzing the presence of the KEGG modules. A total of 41 KEGG modules, belonging to 17 categories, were found in the 324 genomes (Supplementary Figure S2). These KEGG modules were involved mainly in the following categories: central carbohydrate metabolism (*n* = 8), cofactor and vitamin metabolism (*n* = 7), purine metabolism (*n* = 4), other carbohydrate metabolism (*n* = 4), arginine and proline metabolism (*n* = 4), etc. A total of 30 out of the 41 KEGG modules were found in most strains (≥ 95%). The presence of these modules indicates that *L. plantarum* can produce various metabolites, including lysine (M00525), acetate (M00579), inosine monophosphate (IMP, M00048), riboflavin (M00125), and tetrahydrofolate (M00126). And a large proportion of *L. plantarum* strains (*n* = 304, 93.8%) could also produce histidine (M00026), a conditionally essential amino acid, suggesting the existence of heterogeneity.

**Figure 2:**
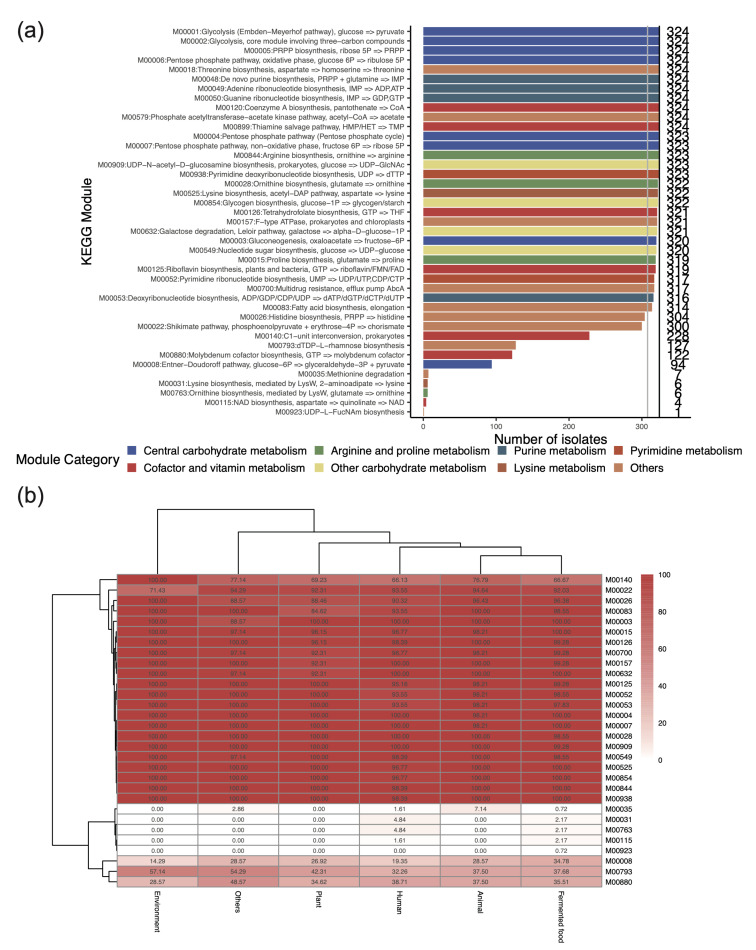
Presence of KEGG modules in the genomes of *L. plantarum*. (**a**) Prevalence of the KEGG modules among the strains. The different colors of each bar correspond to different KEGG categories. The gray vertical line indicates the 95% presence ratio. The numbers on the right represent the total number of strains within the correlated KEGG modules. (**b**) Prevalence of the KEGG modules from various hosts, excluding those modules present in all strains. The positive ratio of different modules in strains from different sources is represented by the numbers in each cell.

We then compared the differences in the KEGG modules between strains from various origins. This revealed that the KEGG modules of human-derived *L. plantarum* were similar to those of *L. plantarum* from animal and fermented food sources. Compared with the environmental samples (100.0%), human-, animal-, and fermented food-derived samples presented lower proportions of C1-unit interconversion (M00140), ranging from 66.1% to 76.8%. Conversely, compared with the environmental samples (71.4%), the human-, animal-, and fermented food-derived samples presented higher proportions of chorismate production (M00022), ranging from 92.0% to 94.6% (Figure 2a). Certain KEGG modules were also present in some strains from human, animal, and fermented food sources and also contained certain KEGG modules (Figure 2b), including methionine degradation (M00035), lysine biosynthesis (M00031), and ornithine biosynthesis (M00763). These findings suggest that human-derived strains may be acquired from fermented foods and animals.

### 3.4. Profile of Glycoside Hydrolases (GHs) Reveals the Ability to Degrade a Variety of Carbohydrates

**Figure 3:**
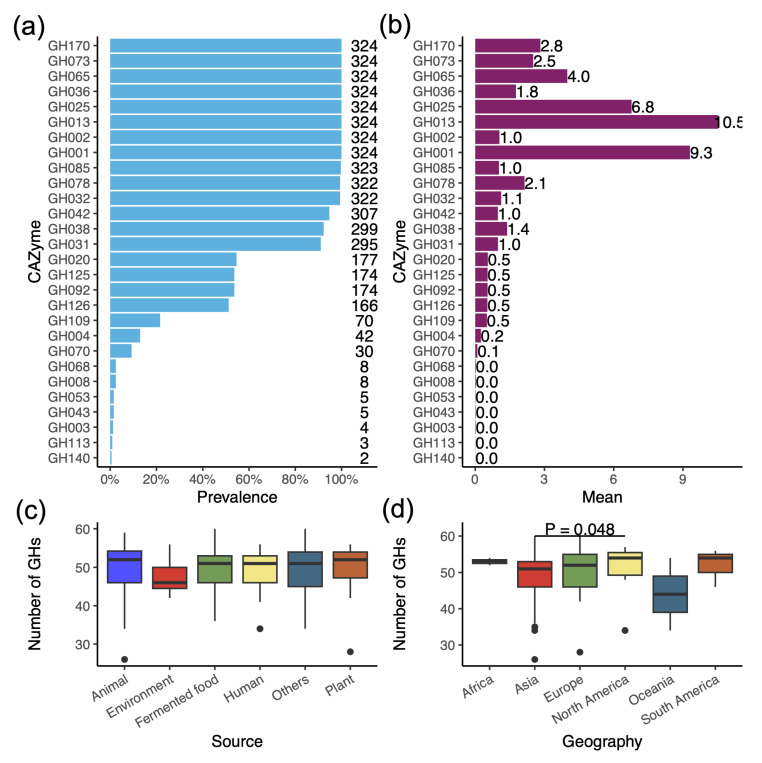
Glycoside Hydrolase (GH) family harbored in *L. plantarum* genomes. (**a**) Prevalence of GH families in the *L. plantarum* genomes. The numbers on the right indicate the total number of strains in correlated GH. (**b**) The average copy number of each GH family in the *L. plantarum* genomes. (**c**) The number of GHs in strains from different sources. (**d**) The number of GHs in strains from different continents.

*L. plantarum* strains typically possess many genes encoding GHs, which help them to adapt to diverse environments. An annotation of the CAZymes revealed that *L. plantarum* can encode at least 28 different GH families, 11 of which are present in most strains (≥95.0%) (Figure 3a). Notably, some GH families are not only prevalent in *L. plantarum* but also have multiple copies, which help to degrade carbohydrates (Figure 3b). For example, eight GH families were present in all strains: GH1 (β-glucosidases and β-galactosidases), GH2 (β-galactosidases, β-mannosidases, exo-β-glucosaminidases), GH13 (α-glucosides), GH25 (lysozymes), GH36 (α-galactosidases and α-N-acetylgalactosaminidases), GH65 (phosphorylases), GH73 (α-N-acetylglucosaminidases), and GH170 (6-phospho-N-acetylmeramidases). The average number of copies of the eight GH families in the 324 genomes ranged from 1.0 to 10.5. Interestingly, each genome contained an average of 10.5 copies of GH13 and 9.3 copies of GH1.

For each genome, the average number of GH families across the 324 genomes was 49.5, with a range of 26 to 60. There was no significant difference among the strains from different sources. Additionally, the strains that are isolated from North America exhibited a higher number of GHs than those from Asia (Figure 3c,d)

### 3.5. Profile of Protease Types Reveals Their Ability to Degrade or Modify a Variety of Proteins

**Figure 4:**
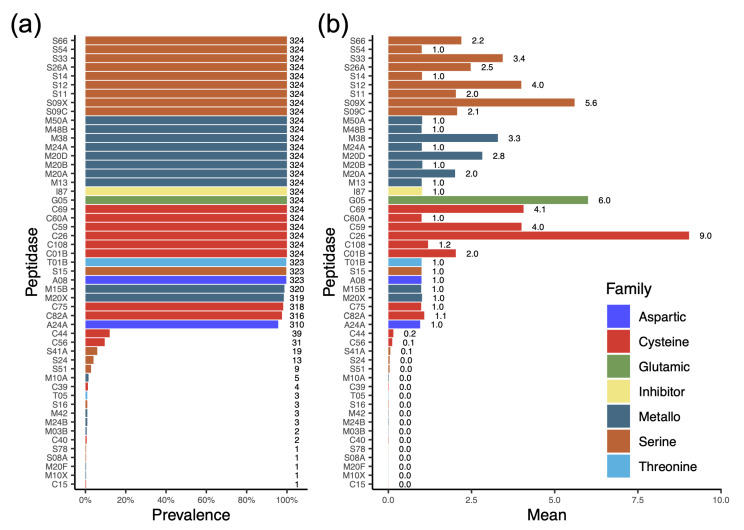
Protease families and inhibitors harbored in the *L. plantarum* genomes. (**a**) Prevalence of protease families and inhibitors in the *L. plantarum* genomes. The numbers on the right represent the total number of strains in correlated peptidase. (**b**) The average copy number of each protease family/inhibitor in the *L. plantarum* genomes.

In microbes, peptidases are crucial enzymes that degrade proteins to acquire nutrients, process essential cellular components, and often play key roles in virulence, the stress response, and environmental adaptation. A total of 51 different protease families distributed across seven protease types were detected in the *L. plantarum* genomes (Figure 4a). Metallo-, serine- and cysteine-peptidases were common members of the *L. plantarum* genome. Among these three peptidases, there were eight, nine, and six families found in the metallo-, serine-, and cysteine-type peptidases, respectively. These may be essential components of the *L. plantarum* genome. Moreover, the inhibitor I87 was detected in all *L. plantarum* genomes. Interestingly, the average number of copies of C26 per *L. plantarum* genome was 9.0 (Figure 4b), which is often involved in the turnover of folyl poly-gamma-glutamates.

### 3.6. The Antimicrobial Peptide (AMP) Facilitates the Antimicrobial Properties of L. plantarum

**Figure 5:**
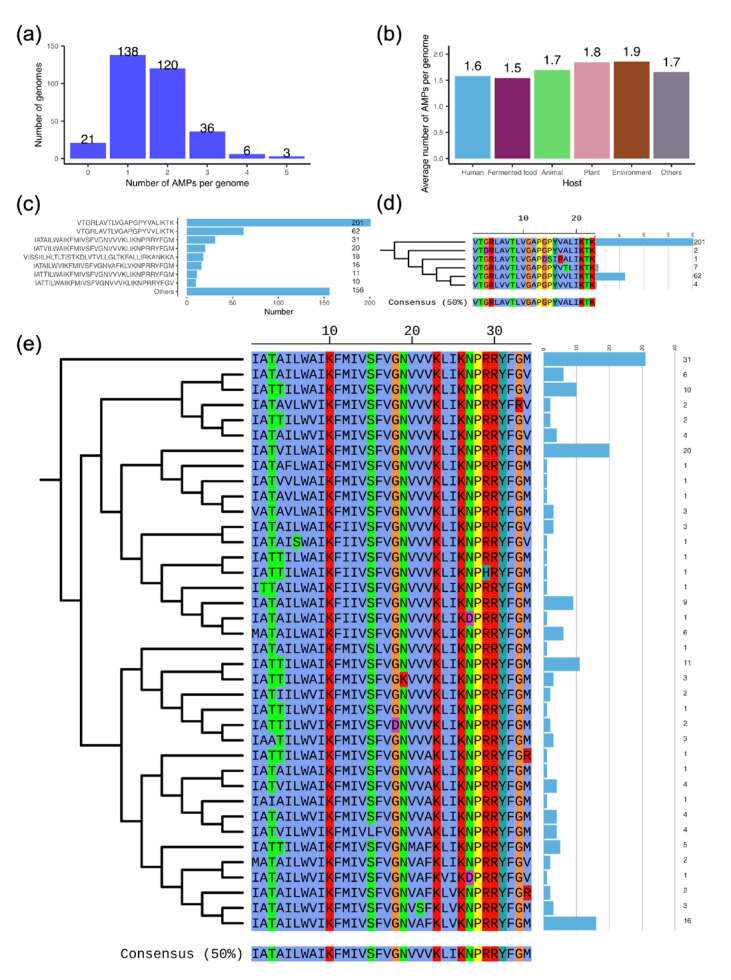
Antimicrobial peptides (AMPs) harbored in *L. plantarum* genomes. (**a**) Number of AMPs per genome. (**b**) Average number of AMPs per genome from various sources. (**c**) Number of the most common AMP sequences. (**d**–**e**) Phylogenetic tree of two clusters of AMP sequences. The sequence of each branch was labeled on the tree, and the number of each AMP was shown in the blue bar. The numbers on the right represent the total number of AMP sequences (**c**–**e**). Different amino acids were colored according to their chemical properties in (**d**–**e**).

*L. plantarum* can produce AMPs that inhibit the growth of other bacteria. Therefore, we predicted which AMPs *L. plantarum* may harbor. AMP sequences were found in a total of 303 out of 324 genomes (93.5%, Figure 5a). On average, each genome from different origins contained an average number of 1.5 to 1.9 AMP sequences (Figure 5b). These AMPs belong to 77 different sequence types. The more common AMP sequence type was “VTGRLAVTLVGAPGPYVALIKTK”, which was found in 201 strains, and its mutant A18V, which was found in 62 strains (Figure 5c). To further verify the novelty of the 77 AMPs, we compared them against the known AMPs (*n* = 44,406). These results indicated that only one AMP sequence from *L. plantarum* SRCM100995 was identical to a partial sequence (50/62) of an existing AMP (L12A07947) recorded in the LAMP2 database. The remaining sequences exhibited identities ranging from 23.3% to 96.0%. Notably, the most prevalent AMP (*n* = 201) demonstrated an identity of no more than 70.0% when compared to the known AMPs. These findings indicate that these AMPs are commonly carried by *L. plantarum*. Furthermore, we clustered these AMP sequences on the basis of a cut-off of 70.0% identity, forming 31 clusters. To further explore the sequence differences within the clusters, we constructed phylogenetic trees of AMPs from the same cluster and predicted their antimicrobial activity. The results revealed that, of the cluster represented by “VTGRLAVTLVGAPGPYVALIKTK” (*n* = 201), the variant A18V (*n* = 62) exhibited stronger antibacterial activity than the others (Figure 5d and Supplementary Table S3). In the cluster represented by “IATAILWAIKFMIVSFVGNVVVKLIKNPRRYFGM” (*n* = 31), a number of 38 different AMP sequence types were found (Figure 5e). Among these mutations, the main mutation types were associated with a certain degree of decrease in antimicrobial activity.

### 3.7. Plasmids Mediate the Transmission of Antibiotic Resistance Genes Within the Lactobacillaceae Family

**Figure 6:**
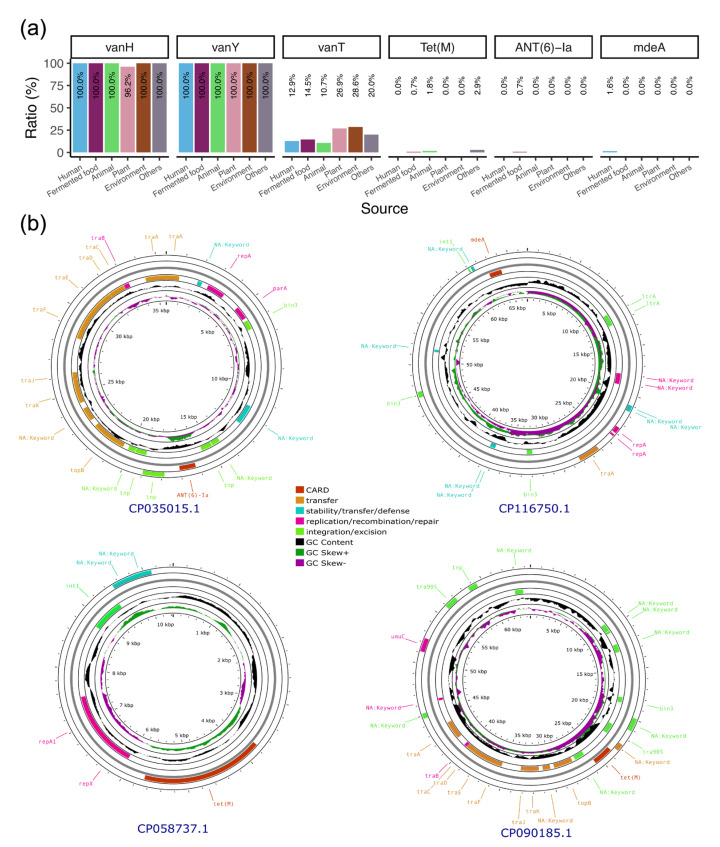
Antibiotic resistance genes (ARGs) that are carried by the *L. plantarum* strains and related plasmids. (**a**) Prevalence of ARGs in the strains from various sources. (**b**) The genetic structure of ARG-carrying plasmids. Note: The plasmids in *L. plantarum* A8 and *L. plantarum* J50 exhibited the same length with only two mismatches (6261/6263) and an ANI value of 0.990.

As probiotics, biosafety is an important aspect that should be considered. Therefore, we first scanned the genomes for virulence factors and found no positive hits. We then explored the ARGs harbored in the genome and identified a total of six ARGs. Two of these (*vanH* and *vanY*) were found in almost all strains, except for those from the plant-related samples, of which the percentage of *vanH*-positive strains was 96.2% (Figure 6a). Another ARG, *vanT*, which confers vancomycin resistance, was present in 10.7% of the animal-derived strains and in 28.6% of the environmental strains. In addition to the vancomycin resistance genes, the *Tet(M)*, *ANT(6)-Ia*, and *mdeA* genes, which mediate tetracycline, aminoglycoside, and multidrug resistance, respectively, were also identified in these strains.

To determine whether these genes are mobile, we analyzed their associations with phages and plasmids. The results revealed that three plasmids carried the *Tet(M)* gene. Among these, the plasmid in *L. plantarum* ST could be transferred within the *Lactobacillaceae* family, as could the plasmid carrying the *ANT(6)-Ia* gene in *L. plantarum* 12_3. The plasmid carrying the *mdeA* gene in *L. plantarum* MWLp-12 could also potentially undergo transfer (Figure 6b and Table 2). We also identified prophage sequences in the genomes, finding an average of one to seven fragments per genome belonging to the genus *Duplodnaviria* (Supplementary Table S4). Additionally, no ARGs were detected in the prophage fragments.

## 4. Discussion

In this study, we conducted a comparative genomic analysis of 324 complete *L. plantarum* genomes. The key findings of this study are as follows: (1) the strains were distributed unevenly from various origins; (2) *L. plantarum* may be able to produce some beneficial metabolites for hosts; (3) there was a high diversity of CAZymes and proteases in the *L. plantarum* genomes; (4) a large proportion of *L. plantarum* could produce AMPs as probiotics; and (5) the plasmid-mediated transmission of ARGs within the *Lactobacillaceae* family requires more attention in the future.

Our research revealed that *L. plantarum* has a total of 2403 core genome genes, which is higher than that of other *Lactobacillus* species, as reported [41,42]. These findings suggest that these genes are important for the survival of *L. plantarum*. Notably, among these core genes, 12.3% of these core genes have unknown functions, and their role in the survival of *L. plantarum* requires additional research, such as the use of the CRISPRi system [43].

The construction of phylogenetic trees can help us to better understand how different strains of the same species are transmitted from various sources. Previous evolutionary tree analyses of 54 *L. plantarum* strains did not reveal a close connection between the strains and their origins [12]. Our more extensive analysis similarly uncovered no inherent connections, indicating that *L. plantarum* sourced from diverse origins is disseminated via a multitude of pathways. For instance, plant-derived *L. plantarum* can be further transmitted to humans via the fermentation enrichment of fermented foods. However, the ability of colonization of the strains from fermented foods needs to be studies in the future.

Microorganisms in the gastrointestinal tract can synthesize essential amino acids de novo to maintain amino acid homeostasis in the host [44]. We found that *L. plantarum* can synthesize several essential amino acids for humans, including threonine, arginine, and lysine, which are crucial for maintaining the body’s amino acid balance. Additionally, *L. plantarum* can also synthesize histidine, a conditionally essential amino acid that helps maintain nutritional balance in early infancy [45]. The supplementary of essential amino acids to hosts may bring benefits to hosts.

In addition to essential amino acids, *L. plantarum* can also synthesize various beneficial compounds. For example, it can produce IMP due to the presence of M00048. IMP enhances the flavor of fermented foods as a flavoring substance [46]. The genome of *L. plantarum* has also been found to metabolize and produce acetic acid, which inhibits the proliferation of pathogenic bacteria during fermentation and aids in enhancing the resistance of the host to pathogenic infections and regulating the immune system [47]. The presence of the thiamine salvage pathway results in the production of thiamine (vitamin B1). Thiamine is essential for all living organisms because its active form, thiamine pyrophosphate, is an indispensable cofactor for enzymes that are involved in amino acid and carbohydrate metabolism [48].

An analysis of the CAZymes in *L. plantarum* indicates that it is a highly adaptable heterotroph that can utilize various complex carbohydrates as energy sources. For example, GH13 and GH1 are essential for the metabolism of complex carbohydrates [49], and multiple genes from these families increase the degradation ability. Additionally, the presence of various proteases not only helps *L. plantarum* to utilize different proteins effectively but also helps the host to digest and absorb proteins. These results suggest that *L. plantarum* has strong capabilities for utilizing polysaccharides and proteins, which endows it with significant environmental adaptability and assists the host in the digestion and absorption of nutrients.

As a probiotic, research has shown that its main mechanism of action is producing antimicrobial peptides. We found that *L. plantarum* primarily produces two types of antimicrobial peptides, represented by “VTGRLAVTLVGAPGPYVALIKTK” and “IATAILWAIKFMIVSFVGNVVVKLIKNPRRYFGM”. These two AMPs are not recorded in any antimicrobial peptide database. Furthermore, the antimicrobial activity of these mutants exhibits some degree of variability, which could impact strain selection during use.

We also analyzed the virulence factors and antibiotic resistance genes carried by the bacterium and found that our results are consistent with those of previous reports, indicating that there are no known virulence genes in its genome [9]. However, unlike previous studies, we discovered several genes that may undergo horizontal gene transfer. These genes are carried by plasmids that can be transferred within the *Lactobacillaceae* family.

The present study also has a limitation. While the in silico genomic analyses of *L. plantarum* offer insights into the genetic diversity, metabolic function, and antimicrobial properties of this species, this study lacks additional experimental validation. Specifically, the mobility of the plasmid-carrying ARGs, the biological activity of AMPs, and the metabolites require further investigation. These findings need to be verified to serve as a guide for future applications.

## 5. Conclusions

In this study, we conducted a comprehensive analysis of 324 *L. plantarum* genomes sourced from various origins and geographical locations. Our findings indicate transmissions occurring from foods to humans, other than from the environment. Furthermore, its ability to utilize a diverse range of carbohydrates and proteins supports its application in fermented foods. As a probiotic, one of the potential beneficial mechanisms of *L. plantarum* may be the production of various AMPs that inhibit other commensals, which can be considered as an intrinsic characteristic of this species. However, the plasmid-mediated dissemination of ARGs should be closely monitored within the fermented food industry.

## Figures and Tables

**Table 1:** Number of core and accessory genes in 324 genomes.

Category	Cut-Off	Number of Genes
Core genes	99% ≤ strains ≤ 100%	2194
Soft-core genes	95% ≤ strains < 99%	209
Shell genes	15% ≤ strains < 95%	1164
Cloud genes	0% < strains < 15%	8849
Total genes	0% < strains ≤ 100%	12,416

**Table 2:** Molecular characterization of the five ARG-carrying plasmids.

Accession	Strain Host	ARG	Replicon Type	Predicted Mobility	Predicted Host Range
CP035015	*L. plantarum* 12_3	*ANT(6)-I* *a*	rep_cluster_707	Conjugative	*Lactobacillaceae*
CP058737	*L. plantarum* A8	*Tet(M)*	rep_cluster_2119	Non-mobilizable	*Lactobacillaceae*
CP090185	*L. plantarum* ST	*Tet(M)*	rep_cluster_167, rep_cluster_707	Conjugative	*Lactobacillaceae*
CP116750	*L. plantarum* MWLp-12	*mdeA*	rep_cluster_1328	Mobilizable	*Lactobacillales*
CP140093	*L. plantarum* J50	*Tet(M)*	rep_cluster_2119	Non-mobilizable	*Lactobacillaceae*

## Data Availability

The genomes included in this study can be found in https://www.ncbi.nlm.nih.gov/datasets/genome/?taxon=1590.

## Supplementary Materials

The following supporting information can be downloaded at: https://www.mdpi.com/article/10.3390/genes16080869/s1, Table S1: Genomic characteristics of 324 strains of *L. plantarum*; Table S2: Number of Glycoside Hydrolases in *L. plantarum* genomes. Table S3: Prediction of minimum inhibitory concentration of AMPs against 34 type strains; Table S4: Prophage fragments in 324 *L. plantarum* genomes; Figure S1: Percentage of strains from different hosts (a) and locations (b) in each cluster in Figure 1. The numbers labeled above each bar represent the total number of strains in each cluster; Figure S2: Heatmap of the presence of KEGG modules in strains from various sources. The hierarchical clustering was based on Euclidean distance and adopted complete linkage. Both rows and columns were clustered.

## Abbreviations

The following abbreviations are used in this manuscript:

AMP	Antimicrobial Peptide
ANI	Average Nucleotide Identity
ARG	Antibiotic Resistance Gene
CARD	Comprehensive Antibiotic Resistance Database
CAZyme	Carbohydrate-Active Enzyme
GH	Glycoside Hydrolase
IMP	Inosine Monophosphate
KEGG	Kyoto Encyclopedia of Genes and Genomes
MGE	Mobile Genetic Element
MIC	Minimum Inhibitory Concentration
NCBI	National Center for Biotechnology Information
