# Supplementary figures and images for: Comparative Genomic Analysis of Lactiplantibacillus plantarum: Insights into Its Genetic Diversity, Metabolic Function, and Antibiotic Resistance
Source: Genes (Basel). 2025 Jul 24;16(8):869. doi: 10.3390/genes16080869 (PMC12386082; doi:10.3390/genes16080869)

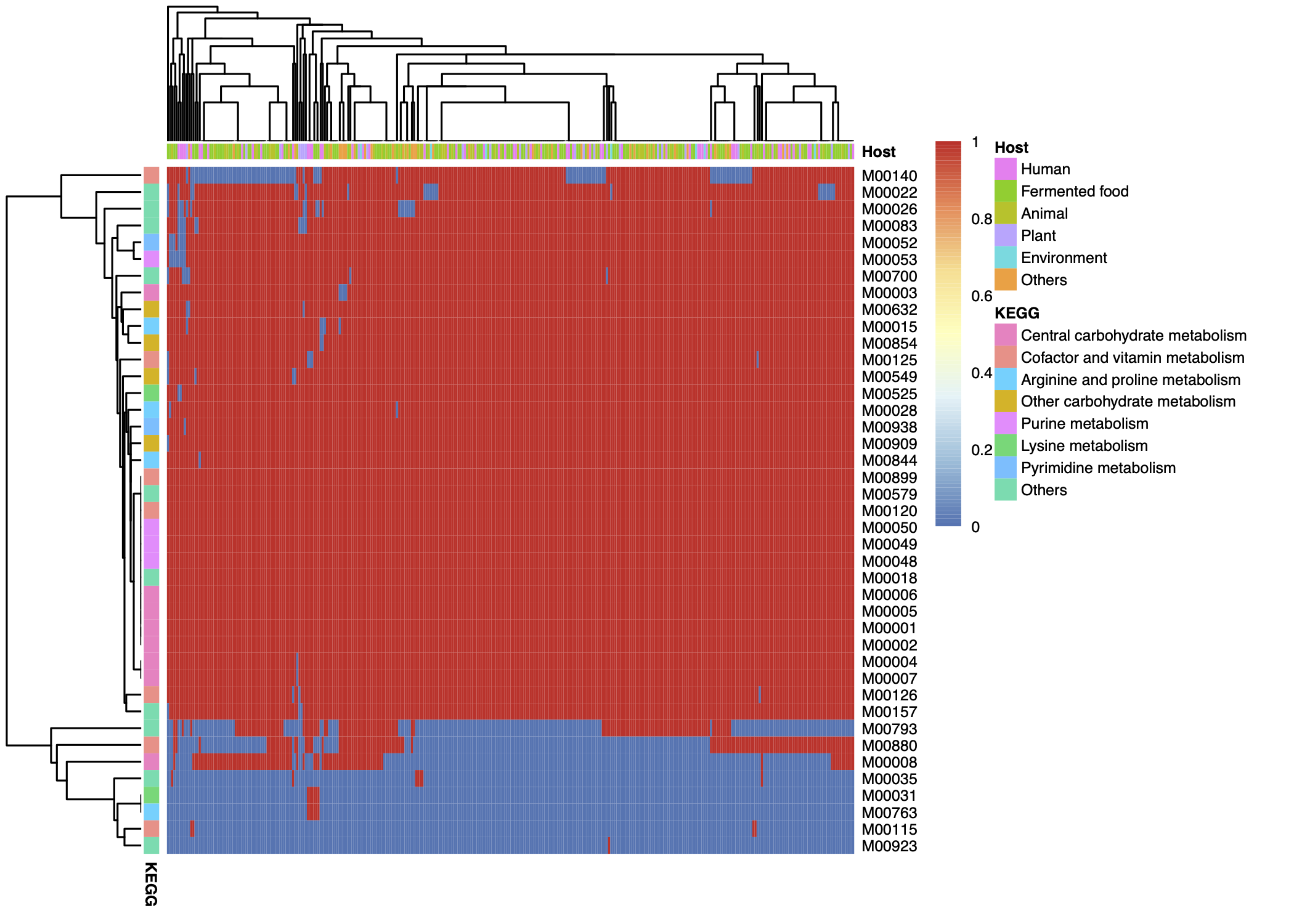

Supplement: Supplementary file 1 [file genes-16-00869-s001.zip › supp/Figure S2.tiff]

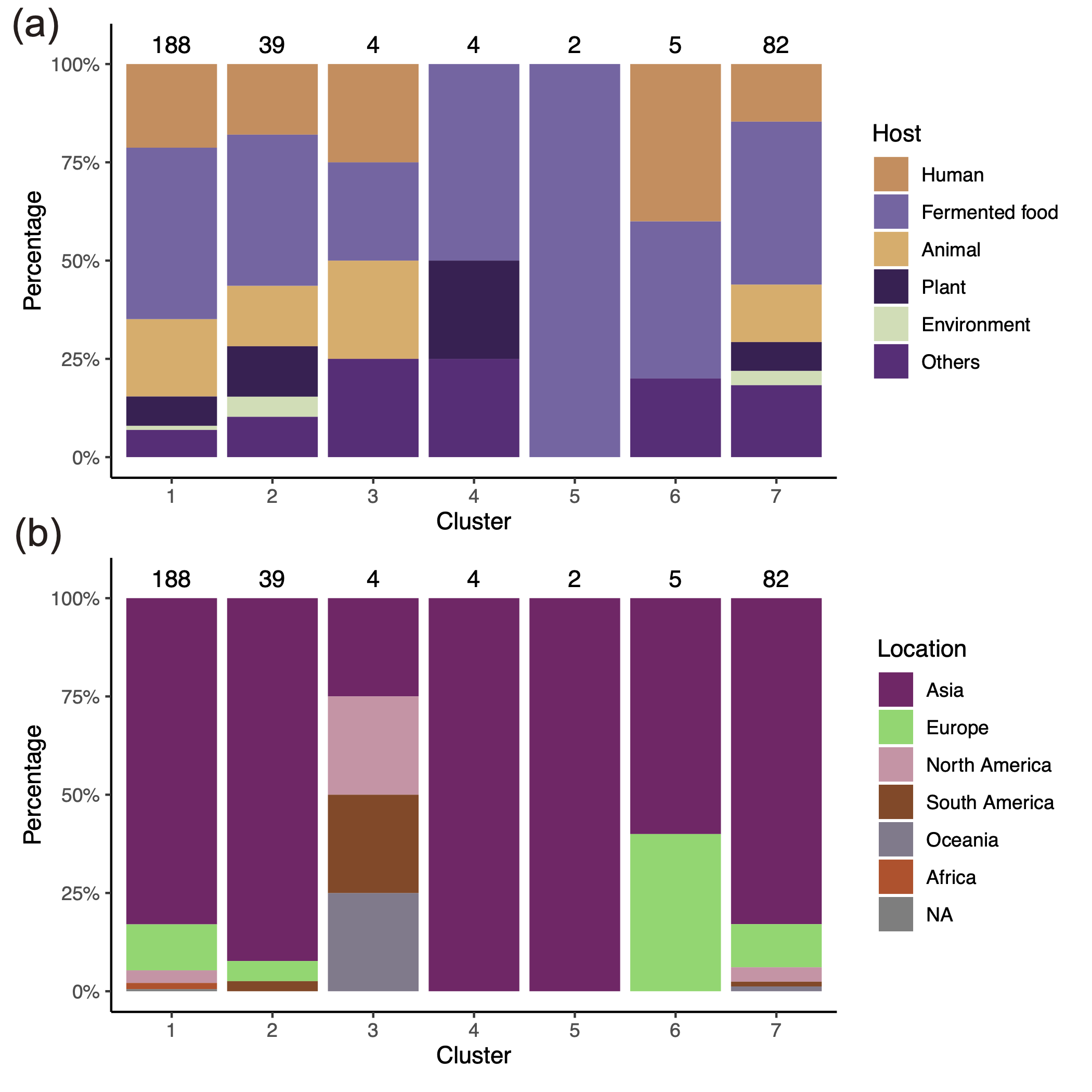

Supplement: Supplementary file 1 [file genes-16-00869-s001.zip › supp/Figure S1.tiff]
